# Supplementary figures and images for: Morphological responses of a temperate intertidal foraminifer, Haynesina sp., to coastal acidification
Source: Front Microbiol. 2025 Jul 10;16:1570629. doi: 10.3389/fmicb.2025.1570629 (PMC12286972; doi:10.3389/fmicb.2025.1570629)

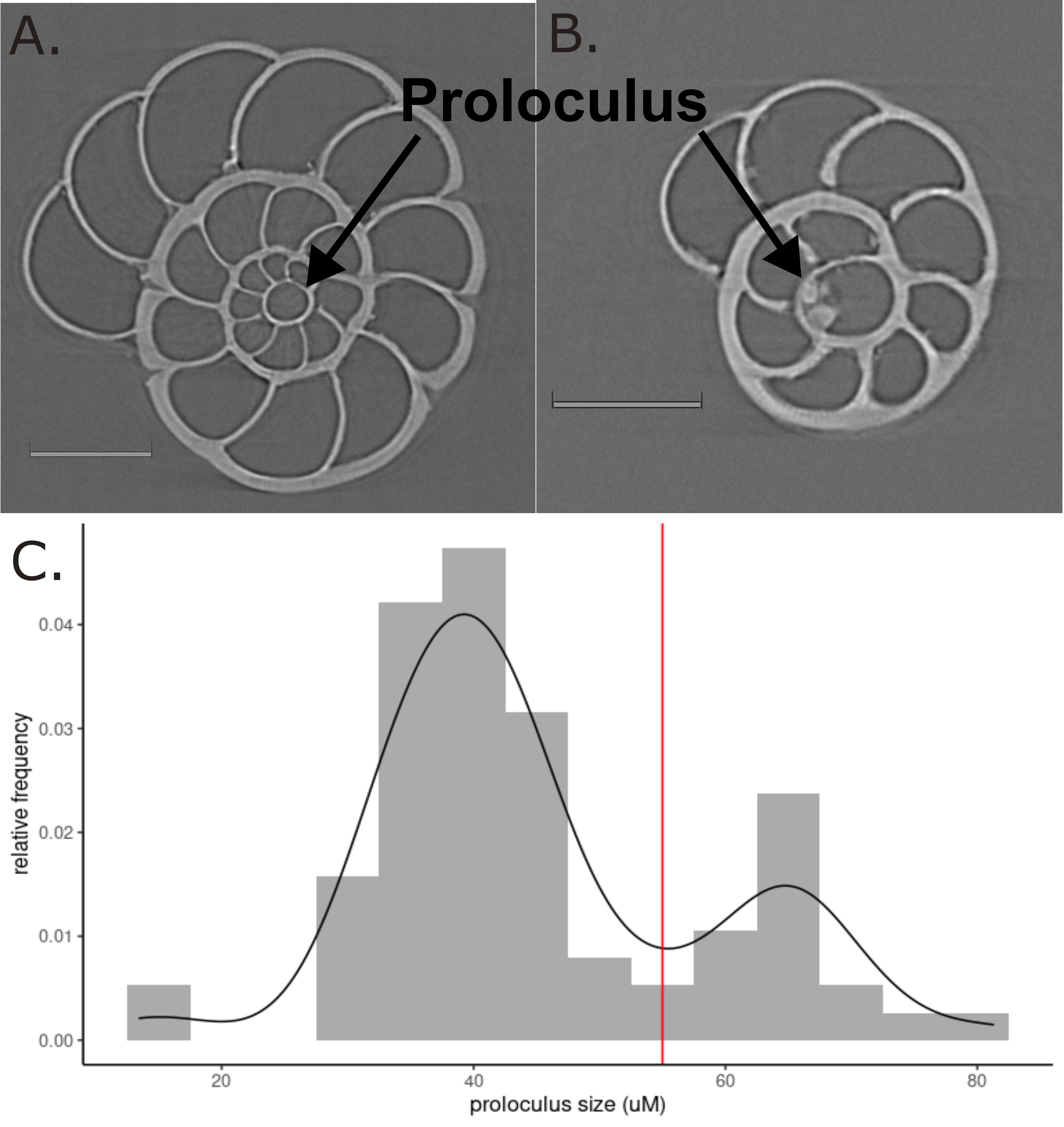

Supplement: SUPPLEMENTARY FIGURE S1 — (A) Cross section of a microspheric test. (B) Cross section of a megalospheric test. Each scale bar represents 100 μm. (C) Histogram of proloculus diameters showing a bimodal distribution. [file Supplementary_file_1.zip › Supplemental Figure S1.jpg]

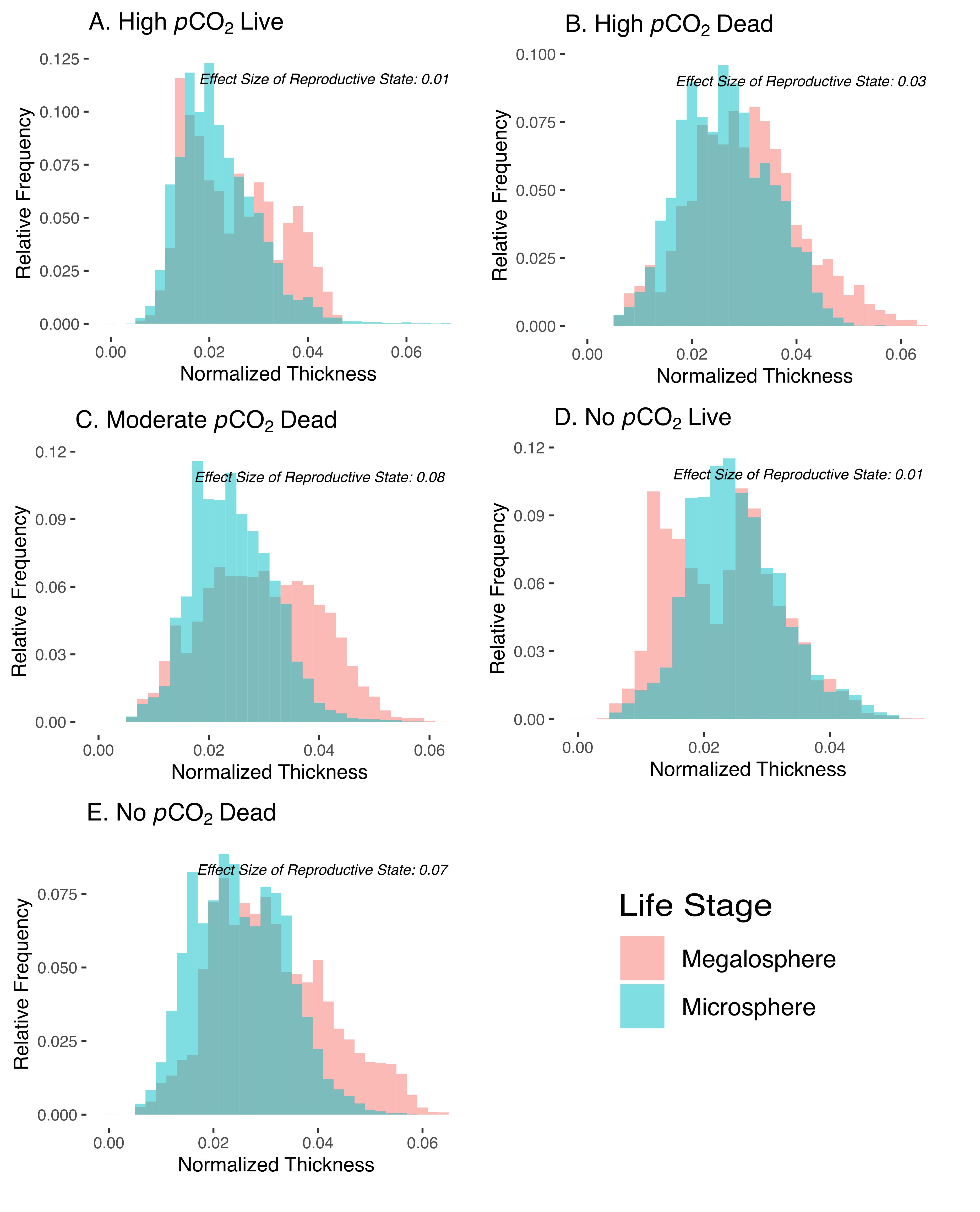

Supplement: SUPPLEMENTARY FIGURE S1 — (A) Cross section of a microspheric test. (B) Cross section of a megalospheric test. Each scale bar represents 100 μm. (C) Histogram of proloculus diameters showing a bimodal distribution. [file Supplementary_file_1.zip › Supplemental Figure S2.jpg]

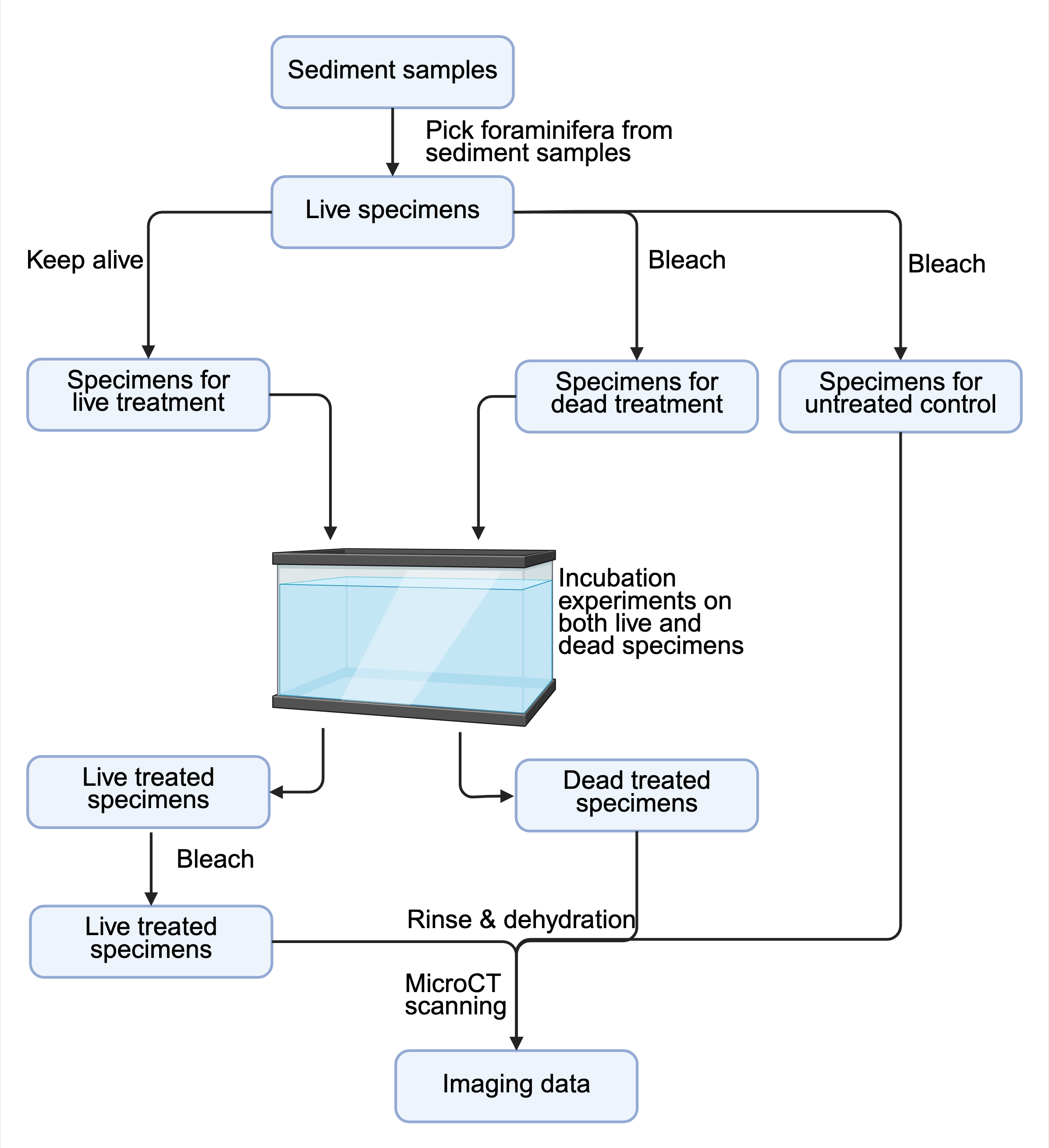

Supplement: SUPPLEMENTARY FIGURE S1 — (A) Cross section of a microspheric test. (B) Cross section of a megalospheric test. Each scale bar represents 100 μm. (C) Histogram of proloculus diameters showing a bimodal distribution. [file Supplementary_file_1.zip › Supplemental Figure S3.jpeg]
